# Our First Christmas Day

**Published:** 1886-12-25

**Authors:** 


### OUR FIRST CHRISTMAS DAY.

A Happy Omen.

By a singular coincidence, which we hail as a happy omen, the first Christmas Day in the life of The Hospital happens to be also its day of publication. We are reminded by this circumstance that all hospitals, founded upon the love of man for man, may be said to date their birth from that morning, so many centuries ago, when a Child of the royal lineage of David was born among the hills of Judæa. Strange seed to have produced such momentous and wondrous fruit!

A Pitiless Civilisation.

At that far-off period the iron heel of Rome was on Judæa, and almost the entire world. Justice, it is true, held proud sway over the awed peoples who bowed themselves before the Imperial Sceptre; but pity and charity had no place among the legions, or among those who led the legions to battle and to victory. Wherever the eagles were carried, there went law and government, but love was a thing unknown. Human life was of no more account than that of the wild beasts, which struggled together in the arena for the pleasure of a spectacle-loving people. The multitudes, from whose ranks our modern hospitals are filled, were no more regarded than were the ravens which picked their bones, as they lay dead on the field of slaughter. The struggle for survival was then at its fiercest. A private soldier who combined unlimited energy of character with a sufficient measure of unscrupulousness, might fight his way to the throne of the Cæsars, and guide the policy of Imperial Rome.

A New Epoch.

Into that iron world, at that ruthless period, came the Founder of Christianity, with words of peace, charity, and brotherhood. Whatever be the distinctive virtues of modern civilisation, they date from that epoch. The true value of man, as man, received enunciation and permanent confirmation then. The new spirit of the time took possession of the noblest minds of the time, and through them permeated society to its lowest depths. That which was before despised became precious. What has been well called by one of our finest modern writers "the enthusiasm of humanity," supplanted in the minds of men the vulgar desire for military conquest and ephemeral glory. Union, not discord; peace, not war; brotherhood, not personal ambition; pity, not pride—these became the watchwords of poets, preachers, and even princes. There came into the hearts of the early leaders of the Church a vast and all-consuming desire to raise up the down-trodden and the helpless, and to form out of separated and dismembered humanity a new society, in which each human being should have his appropriate and recognised place, as a necessary and worthy part of the completed whole.

The New Gospel.

The poor were to be no longer outcasts; the aged and the sick no longer to be cast aside as worthless. Every human being was stamped with the image of God; and such coinage, however it might be defaced, could never lose entirely the superscription which belonged to it of eternal and indefeasible right. The poor and the sick became in the eyes of many even more precious than those who were prosperous and well, as affording opportunities for the display of the virtues which the new faith had exalted to the highest pre-eminence. So that princes of the Church and even kings could be seen laying aside their rich and stately robes, and, girt with coarse garments, washing the feet of the beggars gathered in from the streets and lanes. So widely did this new spirit prevail, that, in countries where Christianity took firm root, poverty and distress could not be endured with patience. Rich lords and princes vied with poor monks and churchmen who should be first in the race of benevolence and brotherly love. Men dying and passing to a final judgment left large endowments for the poor and sick, hoping, by one great act of piety, to atone for a life's misdeeds.

A Superhuman Change.

Who can estimate the vast, the superhuman change which passed over the spirit of mankind during the first few centuries of the Christian era? We live in the midst of benevolence. Every form of human ill has its special sympathisers and its special abodes of relief. Every want which the sick and suffering experience is anticipated and ministered to. Everything which science can devise or art construct is brought to the aid of the poorest and meanest of mankind. Man, woman, and child is alike remembered, and each has a due share of that which is so beneficent to all. So that for the children even toys and bright pictures, and other graceful gifts, flow in upon them in a never-ceasing stream of increasing abundance.

A Bright and Bounteous Christmas.

What a bright and bounteous season is Christmas to all the hospitals, but especially to those in which children spend a few brief but suffering weeks! We are glad to think, too, that at this time of mutual goodwill, even the paupers in their gloomy and monotonous abodes are not forgotten. The rare Christmas cheer of Old England finds its way to them; and on that day at least no one dares say to them, as they ply the weapons of their gastronomic warfare, "Hold! enough." At this season the gifts flow in without stint or limit, so that we are for once under no necessity to plead and urge upon averted ears the claims of struggling institutions. For once, if only for once, our words shall be those of approval and thankfulness that so many in the world have so profound and lasting a regard for noble duty. However severe their necessities, the hospitals are still open, and many of them are filled to their utmost capacity with those who need their aid. Let us be deeply and unceasingly thankful that, in spite of depressed times, narrowing profits, and diminished incomes, we still see our vast and innumerable charitable institutions with their doors wide open, offering a welcome to all that are in poverty and distress. This is the unceasing echo to that strain which sounded through the hills of Judæa so many centuries ago, "Peace upon earth among men of goodwill."

